# Hsp90 dependence of a kinase is determined by its conformational landscape

**DOI:** 10.1038/srep43996

**Published:** 2017-03-14

**Authors:** Qi Luo, Edgar E. Boczek, Qi Wang, Johannes Buchner, Ville R. I. Kaila

**Affiliations:** 1 Department Chemie, Technische Universität München, Lichtenbergstraße 4, D-85748, Garching, Germany; 2 Soft Matter Research Center and Department of Chemistry, Zhejiang University, 310027, P.R. China; 3 Max Planck Institute of Molecular Cell Biology and Genetics, Pfotenhauerstraße 108, 01307 Dresden, Germany

## Abstract

Heat shock protein 90 (Hsp90) is an abundant molecular chaperone, involved in the folding and activation of 60% of the human kinome. The oncogenic tyrosine kinase v-Src is one of the most stringent client proteins of Hsp90, whereas its almost identical homolog c-Src is only weakly affected by the chaperone. Here, we perform atomistic molecular simulations and *in vitro* kinase assays to explore the mechanistic differences in the activation of v-Src and c-Src. While activation in c-Src is strictly controlled by ATP-binding and phosphorylation, we find that activating conformational transitions are spontaneously sampled in Hsp90-dependent Src mutants. Phosphorylation results in an enrichment of the active conformation and in an increased affinity for Hsp90. Thus, the conformational landscape of the mutated kinase is reshaped by a broken “control switch”, resulting in perturbations of long-range electrostatics, higher activity and increased Hsp90-dependence.

The cellular Src kinase, c-Src, is a tyrosine kinase that plays a central role in cell survival and proliferation, and its increased activity has been linked to cancer development^1^,^2^,^3^. The viral Src kinase, v-Src, originally isolated from the *Rous sarcoma* virus, is a close homolog of c-Src, sharing 98% sequence identity with c-Src^4^,^5^,^6^. However, in contrast to the latter, the constitutive activity of v-Src cannot be down-regulated, thus leading to the formation of sarcomas^7^. v-Src is one of the most stringent client proteins of heat shock protein 90 (Hsp90)^8^,^9^,^10^, an ATP-dependent molecular chaperone involved in the folding and maturation of a wide variety of client proteins in eukaryotes^11^,^12^. Hsp90 functions together with specialized co-chaperones, such as the kinase-specific co-chaperone Cdc37, and helps the client proteins to mature and become fully active^13^,^14^,^15^. Remarkably, c-Src kinase is only weakly affected by Hsp90^16^, whereas the Hsp90-Cdc37 complex strongly binds v-Src, protects it from degradation and increases its activity^8^,^10^,^17^.

c-Src consists of a unique domain, followed by the regulatory SH3 and SH2 domains and a flexible linker, connecting the SH2 domain with the highly conserved kinase domain (KD)^2^ (Fig. 1). The catalytically active KD comprises N- and C-terminal lobes with the active site located at their interface^18^. Motion between the two lobes enables the kinase to adopt an open and a closed conformation that is linked to kinase activation. The KD also contains a C-terminal stretch, with residue Y527 that is phosphorylated by the C-terminal Src kinase (CSK). The phosphorylation of Y527 stabilizes the inactive state of c-Src^19^,^20^,^21^, in which the regulatory SH2 domain binds to the phosphorylated Y527^22^,^23^. In addition, c-Src contains another central phosphorylation site that is important for kinase activity^24^, the conserved Y416 residue, which is located within the activation loop (A-loop).

After dephosphorylation of Y527 has taken place, the c-Src activation process is further driven by ATP-binding and phosphorylation of Y416, which trigger large conformational changes in c-Src (Boczek *et al*., unpublished data). Structural studies^23^,^25^ suggest that the inactive state, with the A-loop in a closed folded conformation, blocks substrates from entering the active site, whereas in the active state, the A-loop unfolds, which further opens up the active site for substrate binding. Based on long molecular dynamics simulations it was recently suggested that the inward movement of the C-helix facilitates the formation of a catalytically active state^26^. The X-ray structures of the active and inactive states of c-Src^23^,^25^, further revealed that E310 changes its ion pair from R409 to K295 during this activation process.

ATP-binding near the C-helix triggers the activation of c-Src by large conformational changes^27^. In the inactive state of c-Src, the unphosphorylated Y416 is buried inside the protein, whereas the activation process triggers the exposure of this part. ATP hydrolysis leads to transphosphorylation of Y416 by another c-Src kinase^28^ that is believed to lock c-Src in a catalytically active state^29^,^30^.

Previous studies have indicated that some amino acid substitutions elevate c-Src activity *in vivo* and lead to increased transformation potential^6^,^31^,^32^. Recently, we showed that three single point mutations (R95W, D117N, and R318Q; “3M”) and a deletion of the C-terminal stretch (“ΔC”), significantly increase the activity of c-Src, transforming the kinase into a strong client protein of Hsp90 that mimics the oncogenic v-Src^17^. It was suggested that the perturbation of central interactions near the active site causes the kinase to flicker between activation states, but the detailed underlying molecular principles still remained enigmatic.

Here, we study the intrinsic protein dynamics of the wild type c-Src, and the oncogenic mutant variants v-Src and c-Src3MΔC in order to elucidate the molecular activation mechanism and the key conformational changes, responsible for causing the strong Hsp90-dependence of v-Src. We show by combined molecular simulations and *in vitro* experiments that the wild type and the v-Src-mimicking mutants greatly differ in their activation barriers, Hsp90-dependence, and conformational flexibility.

## Results

### Essential ion pair dynamics in the active site of c-Src

To probe the effects of ATP-binding and Y416 phosphorylation on Src kinase dynamics, we performed microsecond molecular dynamics simulations of c-Src and c-Src3MΔC initiated from the active state. Figure 2 shows the dynamics of the central E310-K295 ion pair and the A-loop, both of which undergo large conformational changes in the activation process, consistent with previous MD simulations^26^. Our MD data suggest that ATP-binding destabilizes the E310-K295 ion pair, which increases the interaction between E310 and R409 of the A-loop. This in turn weakens the interaction between R409 and Y416, and leads to a more solvent-exposed A-loop, rendering Y416 more accessible for intermolecular autophosphorylation by another Src kinase. Upon phosphorylation of Y416 (pY416), we find that E310-K295 is stabilized in comparison to the ATP-bound state, while the A-loop samples partially unfolded states, as indicated by large <*d*_A-loop_> values (Fig. 2b).

In the simulations of c-Src3MΔC, we observe significant dynamical change in the central active site region in comparison to wild type c-Src (Fig. 2d). We find that the E310-K295 ion pair is unstable in c-Src3MΔC in comparison to c-Src, and in contrast to the wild type protein, this destabilization is not ATP-dependent. In addition, c-Src3MΔC shows a more extended A-loop relative to c-Src, most likely due to the flickering of the E310-K295 ion pair. Similarly to c-Src, the phosphorylation of Y416 in c-Src3MΔC stabilizes the closed conformation of E310-K295. However, the *apo*-form of c-Src3MΔC has a higher population of solvent-exposed Y416, which may lead to an increased propensity for autophosphorylation followed by an increase of kinase activity. These results suggest that the introduced 3MΔC mutations disrupt a molecular “control switch” in c-Src that couples the conformational state of the kinase with ATP-binding and Y416-phosphorylation.

**Figure 1:**
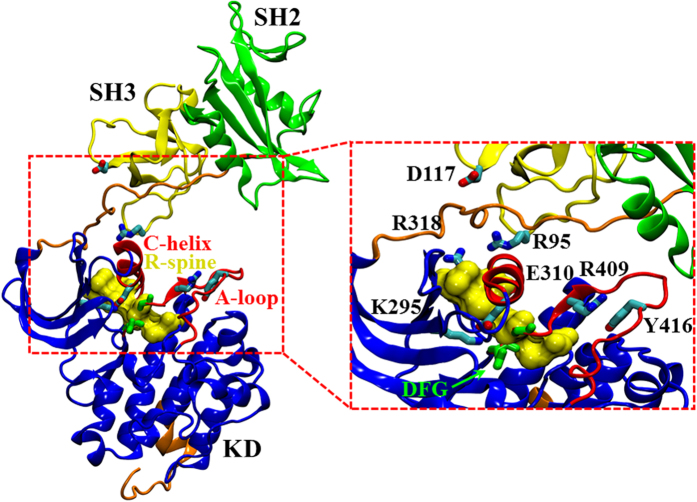
The X-ray structure of the active state of c-Src (PDB ID: 1Y57)^25^. The SH3 domain (in yellow), SH2 (in green), KD (in blue), linker and C-terminal tail (in orange). Inset: the central active site includes the C-helix and the A-loop (in red), 3 M (R95W, D117N, and R318Q), and residues important for kinase activity are depicted in the licorice representation. The DFG-motif are shown in green, and the R-spine regions as a yellow surface.

**Figure 2:**
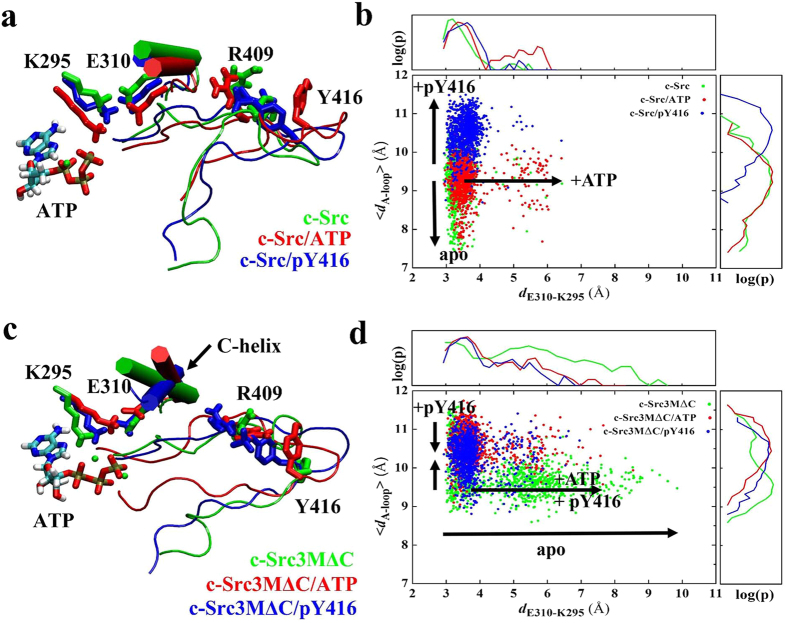
Conformation of the C-helix and the A-loop for (**a**) the wild type and (**c**) the mutant in different states, obtained after 1 μs MD simulations. Conformational dynamics obtained form 1 μs MD simulations of the central E310-K295 ion pair (*d*_E310-K295_) and the extent of the A-loop (<*d*_A-loop_>) are displayed for (**b**) the wild type, and (**d**) the c-Src3MΔC. log(*p*) is the logarithm of the probability distribution for a given reaction coordinate. In wild type c-Src, ATP-binding (in red) destabilizes the E310-K295 ion pair in comparison to the *apo*-form (in green), while phosphorylation of Y416 (in blue) stabilizes the E310-K295 interaction and increases the extent of the A-loop conformation. In c-Src3MΔC the E310-K295 ion pair destabilization is ATP-independent, and the A-loop samples a more extended conformational state. Compared to the wild type Src, the C-helix in the mutants is significantly rotated, showing that the mutations strongly affect the stabilization of the kinase domain.

**Figure 3:**
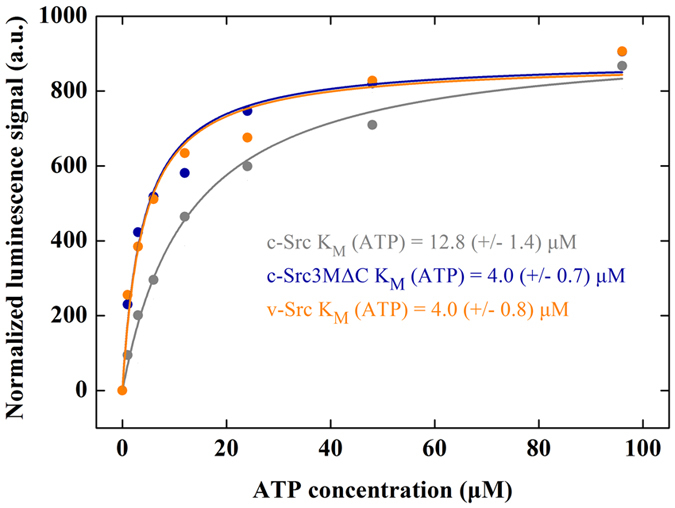
Titration of ATP during kinase activity measurements of c-Src (in grey), v-Src (in orange) and c-Src3MΔC (in blue).

We find that the electrostatic interaction energy of ATP (Supplementary Table S1) with the kinase domain increases in the c-Src3MΔC mutant relative to the wild type, an effect that is likely to result from the destabilization of the E310-K295 ion pair but also by conformational changes in the DFG-motif (D404, F405, G406) and the R-spine region (L325, M314, F405, H384) (Fig. 1), both of which have been suggested to undergo a structural change in the active conformation^33^. For both the wild type and mutant c-Src, we find that ATP-binding and phosphorylation of Y416 destabilizes the R-spine region (Supplementary Fig. S1), in contrast to previous simulations of the isolated KD^30^. A possible reason for this difference might arise from the interaction between the regulatory domains and the KD. To probe the ATP-binding affinities of c-Src and c-Src3MΔC experimentally, we measured the *K*_M_ of the phosphorylation reaction for c-Src, c-Src3MΔC and v-Src *in vitro*. Consistent with our MD data, we find that c-Src3MΔC and v-Src have a three-fold lower *K*_M_ than c-Src with 4.0 μM over 12.8 μM (Fig. 3)^17^.

To study how the large electrostatic perturbations in c-Src3MΔC might shift p*K*_a_ values of active site residues, we performed Poisson-Boltzmann (PB) continuum electrostatics calculations on the structures along the MD trajectories. We find that most residues are in their standard protonation states, shown in Supplementary Table S2. However, we obtain a p*K*_a_ of 6.1 for D404 in c-Src3MΔC, compared to a p*K*_a_ of −3.8 in c-Src, indicating that this residue might reside in a protonated state in the mutant protein, while the protonation probability of the residue in the wild type protein is very low. Such low calculated p*K*_a_ values can arise in PB calculations when the protonation probability for the residue is very low^34^. In contrast, for the wild type protein, the PB calculations suggest that D404 remains deprotonated. It was recently found that D404, which is a part of the DFG-motif, is involved in ATP-binding and its protonation state may also affect the dynamics of the active site residues^35^. We find that protonation of D404 stabilizes the E310-K295 ion pair in 0.5 μs MD simulations (Supplementary Fig. S2), which may result from the weaker interaction between D404 and K295, and thus also contribute to the higher measured ATP-affinity of c-Src3MΔC.

To explore whether the 3MΔC mutations also have an impact on the inactive state, we performed additional simulations based on the crystal structure in the inactive conformation (PDB ID: 2SRC)^23^. Similar to the results obtained for the E310-K295 ion pair in the active state, we find that the E310-R409 ion pair breaks more easily in c-Src3MΔC in comparison to c-Src, suggesting that the dynamics of the inactive state is also strongly affected by the mutations (see Supplementary Information for extended discussion of the inactive state dynamics).

### Ligand-induced global conformational changes

**Figure 4:**
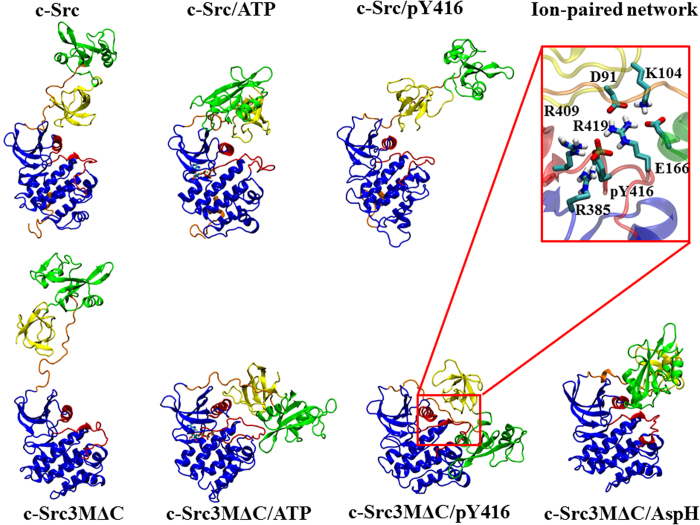
Global structures of wild type and mutant c-Src in different ligand states, obtained after 1 μs MD simulations. c-Src and c-Src/ATP show conformations with a tight connection between the SH3 domain and the KD, while in c-Src3MΔC/pY416 a tight ion paired network (inset), stabilizes the interaction between the SH2 domain and the KD. c-Src/pY416 shows a similar open conformation to c-Src3MΔC, with no direct contacts between the regulatory domains and the KD. c-Src3MΔC/ATP, c-Src3MΔC/pY416 and c-Src3MΔC/AspH have a bent conformation in which the SH2 domain interacts with the KD. The SH3 domain (in yellow), SH2 domain (in green), KD (in blue), linker and C-terminal tail (in orange). The central active site includes the C-helix and the A-loop regions (in red).

In addition to the local structural changes in the active site, we also observe global conformational rearrangements of c-Src and c-Src3MΔC upon ATP-binding and Y416-phosphorylation (Fig. 4). In the ligand-free and the ATP-bound state of c-Src, the SH3 domain interacts with the KD through an ion-paired network. Phosphorylation of Y416 leads to large-scale conformational changes that involve dissociation of the SH3 domain from the central active site. These changes may result from the perturbed interactions between active site residues and R95/E97 of the SH3 domain (Supplementary Fig. S3).

In c-Src3MΔC, we also observe strong alterations of the interaction between the SH3 domain and the KD in the different states (Fig. 4). Compared to c-Src, the wider open conformation of the active state in c-Src3MΔC is likely to result from the R95W and D117N mutations, which destabilize the interaction between the two domains. Moreover, these mutations perturb the interaction between the linker region and the KD, which increases the flexibility of the regulatory domains, resulting in a larger root-mean-square fluctuations (RMSF) of c-Src3MΔC in comparison to the wild type kinase (Supplementary Fig. S4).

When D404 is protonated in c-Src3MΔC, the SH2 domain folds around the KD, with similar structural rearrangements also observed in the ATP-bound state, and when Y416 is phosphorylated in c-Src3MΔC (Fig. 4). These structural changes are stabilized by interactions between arginines in the A-loop and E166 of the SH2 domain and involve an extended ion-paired network (inset of Fig. 4) that stabilizes a solvent exposed A-loop. In contrast, when the SH3 domain and the KD strongly interact, as in the wild type c-Src, this limits the conformational flexibility of the SH2 domain, and prevents the interaction between the SH2 domain and the KD. The electrostatic interactions in the active site thus influence the interaction between the KD and the regulatory domains and lead to the large global conformational changes in c-Src3MΔC (see Supplementary Information for extended analysis on the electrostatic network).

We find that the global dynamics of the ligand-unbound form of c-Src and c-Src3MΔC, extracted from principal component analysis (PCA) of the MD trajectories, show interesting differences (Supplementary Fig. S5). The PCA projects out essential large-scale protein motions by a linear transformation of a covariance matrix of the atomic coordinates. We find that the wild type c-Src dynamics comprise a dominating principal component that involves an *open*-to-*close* motion, enclosing the KD around the regulatory domains. In contrast, in c-Src3MΔC the dynamics involve many contributing modes that comprise transverse motion of the regulatory domains that are coupled to a twist motion of the KD. These differences seem to arise from the perturbed electrostatic interactions between the SH3 domain and the KD in c-Src3MΔC.

### Dynamical similarities between v-Src and c-Src3MΔC

**Figure 5:**
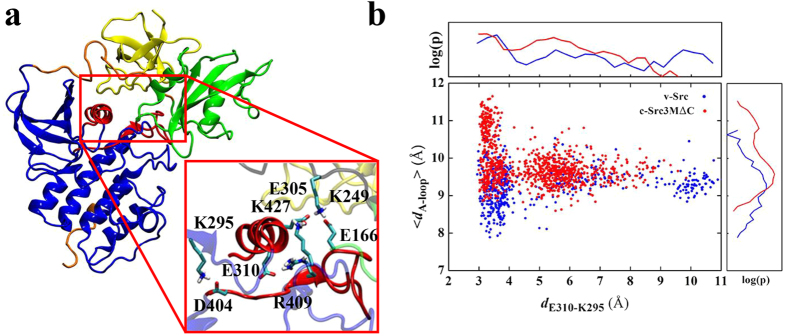
(**a**) Structure and electrostatic network in the active site of v-Src (inset), obtained from 0.5 μs MD simulations. The global conformation of v-Src resembles the ATP and pY416 states of c-Src3MΔC, in which the regulatory domains fold close to kinase domain. (**b**) Conformational dynamics of the central E310-K295 ion pair (*d*_E310-K295_) and the extent of the A-loop (<*d*_A-loop_>) for v-Src (in blue) and c-Src3MΔC (in red) obtained from 0.5–1 μs MD simulations.

In order to compare the dynamics of c-Src3MΔC with v-Src, we performed 0.5 μs MD simulations of a homology model of v-Src, constructed based on the X-ray structure of c-Src (PDB ID: 1Y57). We obtain a similar global structure of the v-Src model as for the ATP and phosphorylated Y416 variant of c-Src3MΔC, in which the regulatory domains fold around the kinase domain. Consistent with the results obtained for c-Src3MΔC, the local ion-paired network around the active site of v-Src is strongly perturbed due to the introduced mutations, which results in the opening of the E310-K295 ion pair and the unfolding of the A-loop (Fig. 5), suggesting that c-Src3MΔC both structurally and dynamically closely resembles v-Src. Interestingly, the E310-K295 ion pair is dynamically even more flexible in v-Src in comparison to c-Src3MΔC, resulting in a stronger interaction between E310 and R409, which is also observed in the inactive state of c-Src. This effect arises from the increased interaction among E305 of the C-helix, K427 of the KD, K249 of the linker region, and E166 of the SH2 domain, which strongly disturbs the C-helix and results in a large displacement of the E310-K295 and E310-R409 ion pairs. These ion pairs undergo a significant conformational transition into an *inactive*-like state in both v-Src and c-Src3MΔC, increasing the E310-K295 distance and decreasing the E310-R409 distance.

### Free energy landscape of the Src activation process

**Figure 6:**
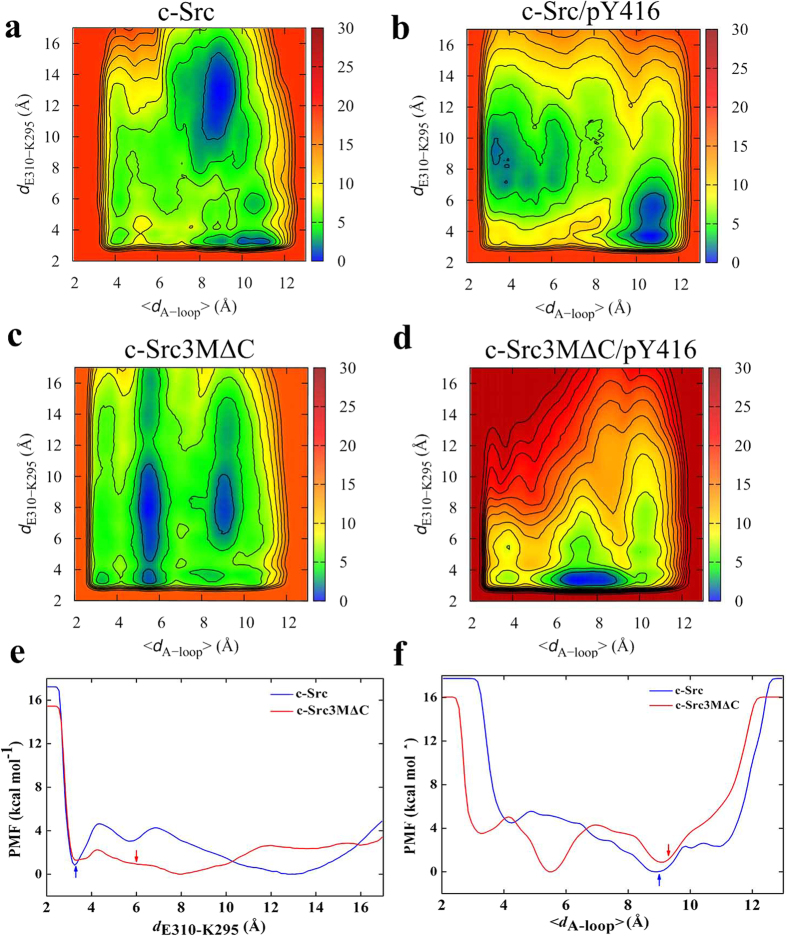
Free energy landscape, Δ*G* (in kcal mol^−1^) for (**a**) c-Src, (**b**) c-Src/pY416, (**c**) c-Src3MΔC and (**d**) c-Src3MΔC/pY416 obtained from 0.5 μs metadynamics simulations with reaction coordinates E310-K295 (*d*_E310-K295_) and the extent of the A-loop (<*d*_A-loop_>). The PMF of unphosphorylated states is projected on *d*_E310-K295_ and <*d*_A-loop_> shown in (**e**,**f**), respectively. The arrows mark the starting positions of *d*_E310-K295_ and <*d*_A-loop_> obtained from MD. The E310-K295 has an activation barrier of *ca*. 4 kcal mol^−1^ in c-Src, while the half-open E310-K295 is thermodynamically stable in c-Src3MΔC.

To probe the dynamics of c-Src on time-scales longer than those accessible by microsecond MD simulations, we performed metadynamics free-energy simulations that fill the potential energy surface by time-dependent potentials. This prevents the system from revisiting already sampled regions of the phase-space, and help the system to overcome large kinetic barriers. The obtained free energy profiles or the potential of mean force (PMF) can be related to the rates by the transition state theory. For the wild type system, we find two minima with E310-K295 in closed (*d*_E310-K295_ ≈ 3 Å) and open (*d*_E310-K295_ ≈ 13 Å) conformations (Fig. 6e), that correlate with partial unfolding of the A-loop (<*d*_A-loop_>≈9 Å), consistent with long MD simulations by Shukla *et al*.^26^. The active and inactive states are nearly isoenergetic, and have a 4 kcal mol^−1^ free energy barrier along the E310-K295 reaction coordinate. Moreover, the partially unfolded A-loop around <*d*_A-loop_>≈9 Å is thermodynamically favored by about 4 kcal mol^−1^ over the folded A-loop with <*d*_A-loop_>≈4 Å (Fig. 6f).

In c-Src3MΔC, the PMF surface (Fig. 6c) suggests that opening of the E310-K295 ion pair and the extent of the A-loop are decoupled, indicating that the activation mechanism differs in the mutant from the wild type kinase. We find that opening of the E310-K295 ion pair has a smooth free energy surface (Fig. 6e), and can sample both open and closed conformations irrespective of the conformation of the A-loop. Moreover, the 1-D projection of the PMF suggests that the half-open state of the E310-K295 ion pair with *d*_E310-K295_ ≈ 8 Å is thermodynamically favored, suggesting that the mutant kinase might always reside in a meta-stable active state. In c-Src3MΔC, the unfolded A-loop, is somewhat less stable in comparison to the wild type system, with an additional minimum at a half-folded conformational state.

To investigate how phosphorylation of Y416 stabilizes the active state, we performed additional 0.5 μs metadynamics simulations to probe its free energy landscape. As shown in Fig. 6b and d, the PMF is considerably altered in the phosphorylated state, trapping both the wild type and the mutant Src kinase in the active conformation. This is consistent with recent observations by Meng and Roux^30^ for the c-Src kinase domain. Interestingly, the A-loop in c-Src3MΔC/pY416 is partially locked in an open state with <*d*_A-loop_>≈8 Å, while c-Src/pY416 samples more folded A-loop conformations with <*d*_A-loop_>≈3 Å.

Taken together, our results suggest that the mutations in the Hsp90-dependent kinase globally reshape the conformational landscape of the kinase leading to rather shallow energy barriers between the different states.

### Less compact structure leads to increased Hsp90-dependence

**Figure 7:**
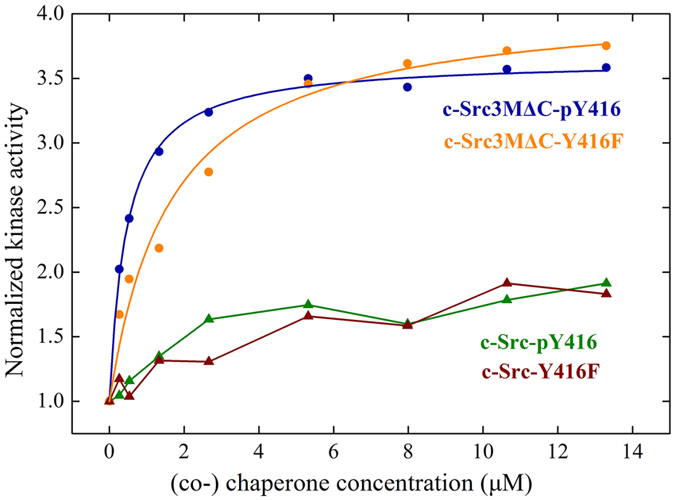
Activation of c-Src and c-Src3MΔC by the Hsp90-Cdc37-complex. c-Src-pY416 (in green) and non-phosphorylatable c-Src-Y416F mutant (in red), c-Src3MΔC-pY416 (in blue), and the non-phosphorylatable Y416F variant of c-Src3MΔC (in orange).

In order to probe how conformational changes may affect the Hsp90-dependence of the kinase, we measured the ability of phosphorylated c-Src and c-Src3MΔC and their corresponding non-phosphorylatable Y416F-variants to become activated by Hsp90 and its co-chaperone Cdc37 by our previously developed activation assay using *in vitro* components^17^ (Fig. 7). We find that c-Src is not activated by the chaperones, regardless of its phosphorylation status. In contrast, c-Src3MΔC shows a high activation potential and its activity is increased about 3-fold upon addition of the chaperones. For the phosphorylated c-Src3MΔC, we obtain an apparent activation constant *K*_A_ for the Hsp90-Cdc37 complex of 0.23 μM. In contrast, for the phenylalanine mutation, in which phosphorylation is not possible, the chaperone concentration for the half-maximal activation of this c-Src3MΔC variant is seven-fold increased (*K*_A_ of 1.64 μM). Interestingly, at higher chaperone concentrations, the two curves converge, which further indicates that although the affinity for the chaperone complex may be lowered for the Y416F mutant, the general activation process remains unaltered.

It has been suggested that Hsp90 recognizes non-native segments in the client proteins^17^,^36^. We therefore analyzed the proportion of structured regions (Table 1) that have been suggested critical for Hsp90-Cdc37 recognition^17^. Although biomolecular force field have a tendency to over-stabilize the amount of folded protein regions^37^,^38^, we nevertheless, observe significant differences between the wild type and c-Src3MΔC/v-Src that may contribute to Hsp90 recognition. For the *apo*-form of c-Src3MΔC, the unstructured protein regions particularly in the β1-β5 strands and the P-loop regions, increase in comparison to the wild type kinase, while the ATP-binding and phosphorylation increase the proportion of unstructured regions for both the wild type and the mutant protein. Moreover, calculations of solvent accessible surface areas (SASA) suggest that the c-Src3MΔC in its phosphorylated state has a more extended structure than the respective wild type protein, for each of the regions that may contribute to the Hsp90-Cdc37 recognition (Supplementary Table S3). Interestingly, we also find that the reduced stability of the E310-K295 ion pair correlates with an enhanced kinase activity and Hsp90-dependence of different c-Src variants (Supplementary Table S4).

## Discussion

We previously showed that c-Src3MΔC is more active than c-Src and in contrast to c-Src, this activity could be increased even further by the combined action of Hsp90 and Cdc37^17^. Here, we aimed to explain these differences by using a combined approach of classical molecular simulations and *in vitro* kinase activity assays.

Our simulations show that in c-Src the unfolding of the A-loop is strictly coupled to ATP-binding due to electrostatic switching within the active site. Consistent with the results of others^39^, our findings suggest that unfolding of the A-loop is likely to increase the propensity of Y416-phosphorylation, which in turn stabilizes the active conformation of the kinase. This suggests that the transition to the active state in c-Src resembles a “control switch”-like mechanism with a strong activating role of ATP-binding and Y416-phosphorylation.

In contrast, c-Src3MΔC can enter its active state in a process that is decoupled from these activating stimuli. Our principal component analysis of c-Src3MΔC indicates increased transitions between inactive and active states and a rather shallow energy landscape in contrast to the open-close motion observed for c-Src. Electrostatic perturbations within the active site suggest an increase in ATP affinity, which we could confirm by *K*_M_ determination *in vitro*. The 3MΔC mutations destabilize the central E310-K295 ion pair, the C-helix and the A-loop by disturbing an electrostatic network located at the active site, and by further breaking the interaction between the active site and the regulatory domains. These effects strongly resemble the dynamics observed in our v-Src model, and these results correlate with the previously reported decreased folding cooperativity of c-Src3MΔC in comparison to c-Src^17^. Moreover, c-Src3MΔC showed a more extended A-loop in comparison to c-Src. This in turn may increase the propensity for phosphorylation and Hsp90-interaction. Thus, these transitions render the mutant Src kinase “conformationally uncontrolled” and more likely to sample states that may interact with Hsp90 and Cdc37.

In general, the conformational landscape of the kinase is reshaped by the presence of the oncogenic mutations, which led to a less compact structure of regions that may specifically interact with Hsp90-Cdc37, especially the β1-β5-strands and the P-loop, supporting previous studies that suggested a correlation between Hsp90 binding and the openness of the kinase fold^17^,^40^,^41^. Moreover, in the pY416-activated state of c-Src3MΔC, we found an even more extended kinase structure that may increase the interaction with Hsp90 and Cdc37. We could confirm this notion *in vitro* and found that phosphorylated c-Src3MΔC has a seven-fold decreased *K*_A_ for Hsp90 as compared to unphosphorylated c-Src3MΔC, suggesting that Hsp90 interacts more strongly with the structurally more extended, phosphorylated client kinase. This result supports the previously suggested hypothesis that Hsp90 may stabilize otherwise meta-stable activated states of the client kinase^17^. A random-sampling of a conformation with higher Hsp90-affinity is also possible, but less likely. Interestingly, we found that at high chaperone concentrations, both phosphorylated and unphosphorylated c-Src3MΔC reach the same level of activation. This indicates that in the unphosphorylated form, the active, Hsp90-interacting conformation of the kinase might be less populated and therefore, more chaperone molecules are needed to trap it.

This study reveals large differences between the activation dynamics in wild type Src kinase and its corresponding oncogenic variant. Our results strengthen the understandings on the relationship between oncogenic kinases and the Hsp90 chaperone machinery, which may form a basis for developing conformation-specific drugs, solely acting on the oncogenic kinase activation pathway.

## Models and Methods

Molecular dynamics simulations were performed based on atomistic molecular models of c-Src and c-Src3MΔC, constructed based on the X-ray structure of c-Src in the active state obtained from the Protein Data Bank (PDB ID: 1Y57)^25^. For the c-Src3MΔC, the C-terminal tail (ΔC) of c-Src was deleted together with mutations of residues R95W, D117N, and R318Q. For both the wild type and the mutant c-Src, three simulation states were explored, with and without bound ATP, and with Y416 modeled in a phosphorylated state. To explore the protein dynamics in the inactive state, models of c-Src and c-Src3MΔC were also constructed based on the X-ray structure of c-Src in the inactive state (PDB ID: 2SRC)^23^. We also performed 500 ns MD simulations of c-Src3MΔC with D404 in a protonated state as well as v-Src, constructed from PDB ID: 1Y57 using MODELLER^42^. Each model was solvated in a water box with *ca*. 34 Na^+^/30 Cl^−^ ions, mimicking a 100 mM NaCl concentration. The molecular systems comprised *ca*. 100 000 atoms, and were simulated in an *NPT* ensemble at *T* = 310 K and *p* = 101.3 kPa for 1 μs, with an integration time-step of 2 fs using the CHARMM27 force field^43^, and treating long-range electrostatics with the Particle Mesh Ewald approach. All simulations were performed using NAMD 2.9^44^ and Visual Molecular Dynamics^45^ was used for analysis. All simulation models are listed in Supplementary Table S6.

Metadynamics simulations were carried out to explore the free energy landscape of the c-Src activation process, based on 0.5 μs simulations performed in NAMD 2.9. The potential of mean force (PMF) for c-Src and c-Src3MΔC were calculated starting from the active state, the phosphorylated state and the inactive state respectively, using the distance of E310-K295 (in the active state) or E310-R409 (in the inactive state), and the extension of A-loop as reaction coordinates^46^,^47^. The A-loop reaction coordinate was defined using the average distance of backbone oxygen and nitrogen distances between D413 and T417; N414 and A418; and, E415 and R419, as suggested by Meng and Roux^30^.

Poisson-Boltzmann continuum electrostatics calculations were carried out to estimate p*K*_a_ values of the MD relaxed structures of c-Src and c-Src3MΔC. The protein was described as a polarizable dielectric medium with *ε* = 4 and the solvent was modeled with *ε = *80. The boundary between protein and solvent was defined by the solvent accessible surface area of the protein probed by a sphere of 1.4 Å radius. The protein was described using the CHARMM36 parameters^48^. The linearized Poisson-Boltzmann equation (LPBE) was solved in APBS^49^, and the Monte Carlo sampling of 2^*N*^ proton states for the *N* = 140 titratable residues were performed using Karlsberg 2^50^,^51^.

Principal component analysis (PCA) was carried out with R using the Bio3D package^52^. The atomic coordinates of Cα were used in PCA, which was performed on MD trajectories with 1000 snapshots, saved every 1 ns, for each protein model.

### Kinase activity measurements

c-Src and mutants thereof were expressed and purified as described Boczek *et al*.^17^. To achieve full Y416-phosphorylation, the kinases were incubated with 10 mM ATP and 5 mM MgCl_2_ overnight and subsequently purified using a PD-10 column (GE Healthcare, München, Germany). For activity measurements of c-Src variants, 320 nM of the respective kinase were incubated at 30 °C for 30 minutes in Src-buffer (40 mM Tris-HCl, 150 mM NaCl, 5% Glycerine, 5 mM DTT, 10 mM MgCl_2_, 1 mM MnCl_2_, pH 7.5) supplemented with 40 μM [γ-32P]-ATP with an activity of 0.5 μCi. A tenfold molar excess of acid-denatured enolase (Sigma, St. Louis, USA) was used as a substrate. The reaction was stopped by adding Laemmli buffer and boiling the sample. The samples were separated by SDS-PAGE and transphosphorylation was detected by applying a phosphor image screen onto the gel. The screen was subsequently analyzed using a Typhoon 9200 phosphoimager and the program Image Quant (GE Healthcare, München, Germany). For chaperone-dependent kinase activation, increasing amounts of Hsp90β and Cdc37-S13E were added prior to the transphosphorylation reaction^17^. For *K*_M_-determination, the kinase activity was measured in the presence of increasing amounts of [γ-32P]-ATP.

## Additional Information

**How to cite this article:** Luo, Q. *et al*. Hsp90 dependence of a kinase is determined by its conformational landscape. *Sci. Rep.* **7**, 43996; doi: 10.1038/srep43996 (2017).

**Publisher's note:** Springer Nature remains neutral with regard to jurisdictional claims in published maps and institutional affiliations.

## Supplementary Material

Supplementary Information

## Figures and Tables

**Table 1:** Proportion of unstructured/unfolded protein regions obtained after MD simulations of each model, analyzed using the STRIDE algorithm^53^.

	c-Src	c-Src/ATP	c-Src/pY416	3MΔC	3MΔC/ATP	3MΔC/pY416	v-Src
SH3	51%	62%	59%	57%	51%	68%	59%
SH2	47%	53%	50%	45%	50%	48%	54%
KD	42%	49%	52%	46%	48%	48%	51%
P-loop	**60%**	80%	80%	**100%**	80%	60%	**80%**
C-helix	13%	13%	13%	7%	7%	20%	13%
αC-β4-loop	70%	80%	70%	60%	60%	70%	70%
E-helix	0%	0%	0%	0%	5%	0%	0%
β1-β5-strands	**28%**	40%	40%	**38%**	36%	40%	**36%**

Regions with changes in secondary structure are highlighted in bold.
