# Supplementary material for: Hsp90 dependence of a kinase is determined by its conformational landscape
Source: Sci Rep. 2017 Mar 14;7:43996. doi: 10.1038/srep43996 (PMC5349555; doi:10.1038/srep43996)
Supplement: Supplementary Information [file srep43996-s1.pdf]

Supplementary Information - Hsp90 dependence of a kinase is determined by its conformational landscape

Qi Luo[1,2,a], Edgar E. Boczek[1,3,a], Qi Wang[2], Johannes Buchner[1], Ville R. I. Kaila[1,*]
[1]Department Chemie, Technische Universität München, Lichtenbergstraße 4, D-85748 Garching, Germany
[2]Soft Matter Research Center and Department of Chemistry, Zhejiang University, 310027, P.R. China
[3]Current address: Max Planck Institute of Molecular Cell Biology and Genetics, Pfotenhauerstraße 108, 01307 Dresden, Germany
[a] contributed equally to this work
[*]E-mail: ville.kaila@tum.de

Structure and dynamics of the inactive state of c-Src and c-Src3MΔC

To explore whether the 3MΔC mutations also have an impact on the inactive state, we performed additional simulations based on the crystal structure in the inactive conformation (PDB ID: 2SRC)[1]. Similar to the results obtained for the E310-K295 ion pair in the active state, we find that the E310-R409 ion pair breaks more easily in c-Src3MΔC in comparison to c-Src. Interestingly, however, while the A-loop retains a folded structure in the inactive conformation of c-Src3MΔC, the A-loop unfolds in c-Src, without opening of the E310-R409 ion pair (Supplementary Fig. S6a). The electrostatic interaction networks illustrate how the mutations affect the active site interactions (Supplementary Fig. S6b and S6c). Similar to the simulations of the active state, we find that several residues stabilize the C-helix in c-Src, while the mutations in c-Src3MΔC perturb these interactions and result in a more flexible C-helix. The free-energy simulations of the inactive state further support the notion that the activation barrier for A-loop unfolding in c-Src is lower than in c-Src3MΔC (Supplementary Fig. S7d). Consistent with our MD simulations, we find that the dissociation of the E310-R409 ion pair in c-Src3MΔC has a free energy barrier of *ca*. 2 kcal mol^{-1}, while the same barrier is *ca*. 4 kcal mol^{-1} in c-Src (Supplementary Fig. S7c), indicating that the c-Src3MΔC may more easily undergo transitions between inactive and active states.

The perturbations in the local protein dynamics of the inactive state lead to large conformational changes in the global protein dynamics, as suggested by a PCA of the MD trajectories (Supplementary Fig. S8). However, opposite to the principle components in the active state, the wild type protein involves several contributing low-amplitude modes. The first principal component comprises a breathing-like motion that involves small dynamical changes in both the SH2 and kinase domain. In c-Src3MΔC, however, the first mode has a large amplitude of nearly 80% and results in large *close*-to-*open* transitions between the regulatory domain and the KD, in which the secondary structure remains nearly unchanged.

The rigid structure of the inactive c-Src may result from the phosphorylated Y527 in the C-terminal tail, which is missing in ΔC. The phosphorylated Y527 forms strong interactions with arginines R155 and R175 of the SH2 domain (Supplementary Fig. S9), which has also

been observed in previous MD simulations[2]. The proportion of folded structures in c-Src decreases by *ca*. 10% during the 1 μs MD trajectory, while the protein folds remains stable in c-Src3MΔC. This difference may result from the strong interactions between the regulatory and kinase domain that imposes strain on the protein.

Extended analysis on how mutations induce electrostatic perturbations in active site dynamics

In order to quantify the difference in kinase dynamics induced by ATP-binding and phosphorylation in c-Src and in c-Src3MΔC, we analyzed the structure and electrostatic interactions of the central active site region (Supplementary Fig. S3 and Supplementary Table S5). We find that the active site residues form an extended electrostatic network, which may be involved in triggering the c-Src activation process. The C-helix has a large dipole moment, with E305/E310 at one end and K315/K316/R318 at the other end of the helix. The dynamics of this helix can thus be perturbed by surrounding charged residues, which are involved in mediating long-range couplings[3]. We find that the root-mean-square fluctuation (RMSF) of the C-helix is reduced upon ATP-binding (Supplementary Fig. S4), which may result from the attraction between the ATP:Mg^{2+} complex and conserved charged residues that form a tight ion paired network. In the ATP-bound state, E97 undergoes a significant movement and forms an ion pair with R409, while breaking the interaction with K315. This structural re-arrangement is further likely to stabilize the C-helix.

Upon phosphorylation of Y416, the strong ion pair between R409 and pY416 weakens the interaction between E310 and R409, which results in stabilization of the E310-K295 ion pair (Fig. 2b, main text). This in turn destabilizes the C-helix, as indicated by the increase in the RMSF of this region (Supplementary Fig. S4). In addition, pY416 strongly attracts arginines in the A-loop and its immediate surroundings, forming tight ion pairs, consistent with recent findings by Meng and Roux[4]. This further leads to a partial unfolding of the A-loop.

In c-Src3MΔC, the R95W and R318Q mutations break the electrostatic network in the active site and decrease the dipole moment of the C-helix (Supplementary Fig. S3d and Supplementary Table S5), which contributes to the destabilization of the C-helix. This in turn weakens the interaction between K295 and D404, which may further contribute to the flickering of the E310-K295 ion pair.

In contrast to c-Src, ATP-binding in c-Src3MΔC stabilizes the E310-K295 ion pair and the C-helix, which results from a partially recovered ion paired network between ATP and the surrounding charged residues in the active center (Supplementary Fig. S3e). The MD simulations further suggest that phosphorylation of Y416 in c-Src3MΔC recovers some of the electrostatic network observed in c-Src and partially compensates for the conformational perturbations caused by the mutations. Three arginines form tight ion pairs with pY416, which stabilizes the E305-R409 and D258-K315 interactions.

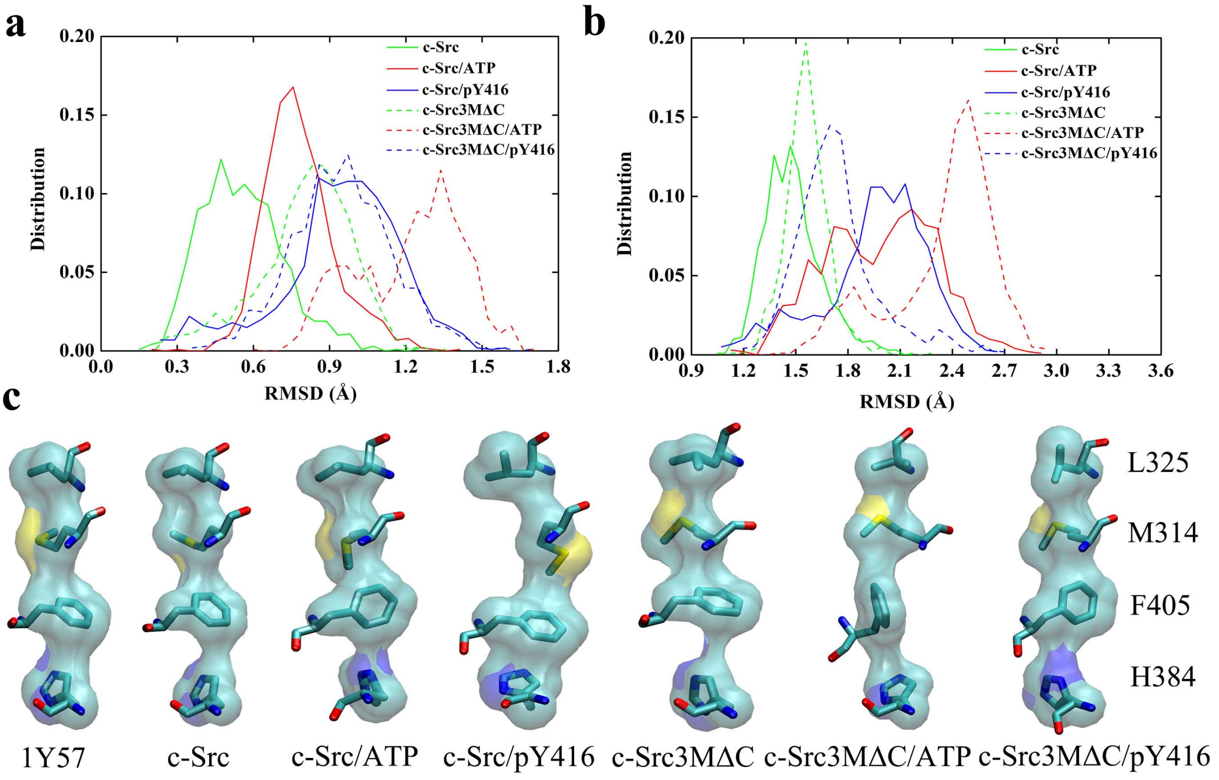

Supplementary Figure S1. (a) Distribution of RMSD of the backbone of R-spine. The backbone RMSD for the inactive state (PDB ID: 2SRC) is 1.30 Å. (b) RMSD Distribution of the side chain conformation in the R-spine. The side chain RMSD for the inactive state (PDB ID: 2SRC) is 2.41 Å. (c) Conformations of R-spine obtained after 1 μs MD simulations, and compared to the crystal structure (PDB ID:1Y57). The R-spine fluctuates more in the ATP-bound and phosphorylated states due to the interaction between the regulatory domains and the KD.

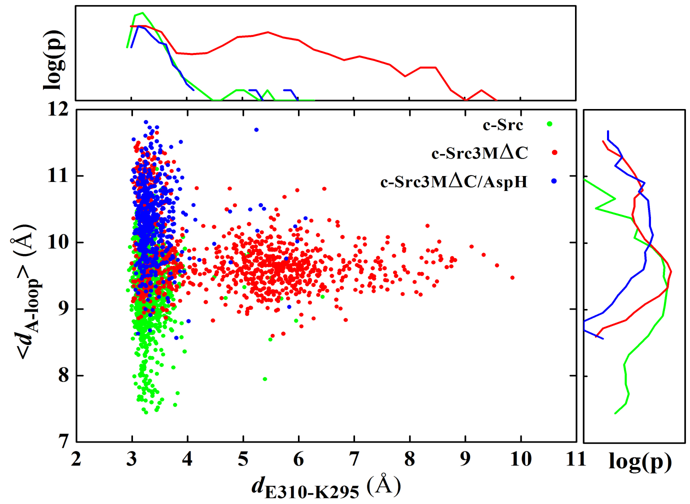

Supplementary Figure S2. Conformational dynamics of the central E310-K295 ion pair ($d_{E310\text{-}K295}$) and the extent of the A-loop ($<d_{A\text{-loop}}>$). log(p) is the logarithm of the probability

distribution for a given reaction coordinate. Protonation of D404 (in c-Src3MΔC/AspH, in blue) stabilizes the E310-K295 ion pair.

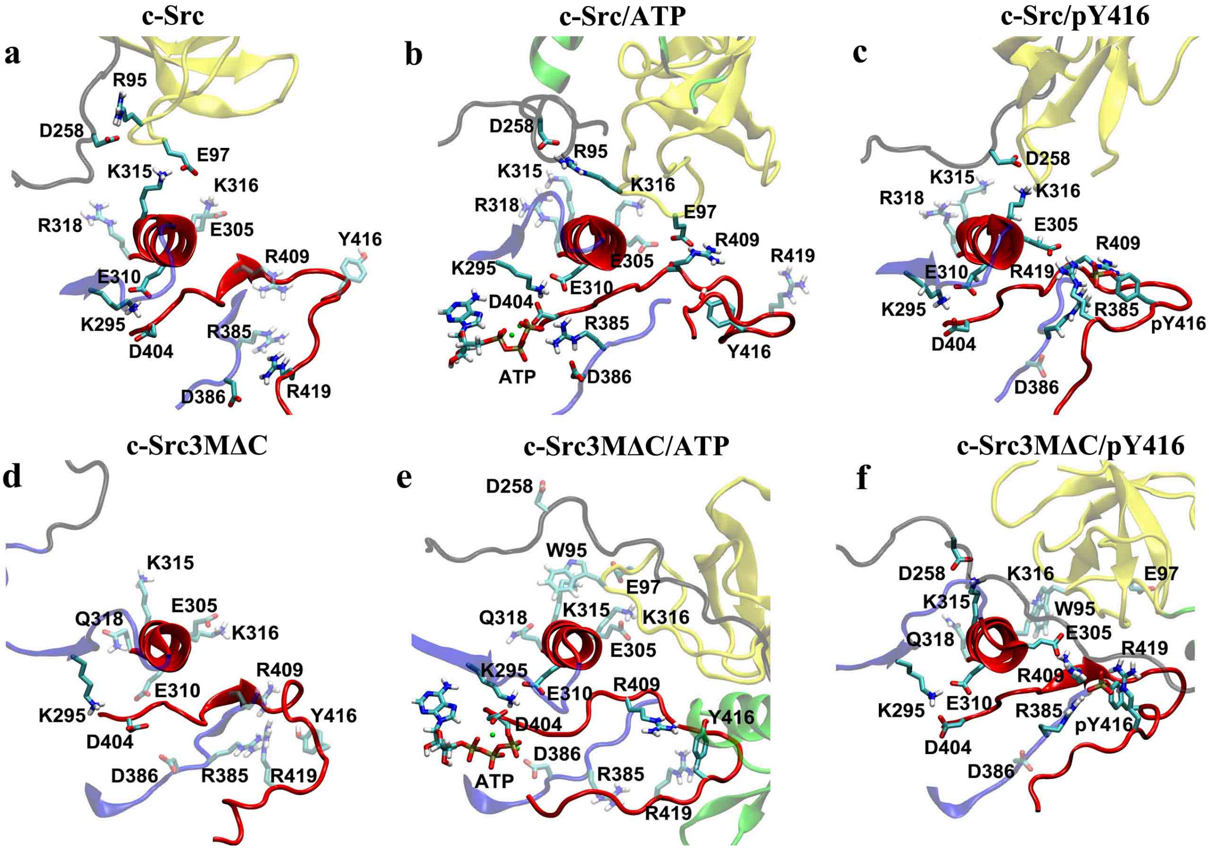

Supplementary Figure S3. Electrostatic network in the active site of c-Src and c-Src3MΔC models obtained after 1 μs MD simulations. The C-helix, A-loop, and residues involved in forming ion pairs are shown in opaque, while other residues are shown in transparent colors. The SH3 domain (in yellow), SH2 (in green), KD (in blue), linker and C-terminal tail (in black), C-helix and A-loop (in red).

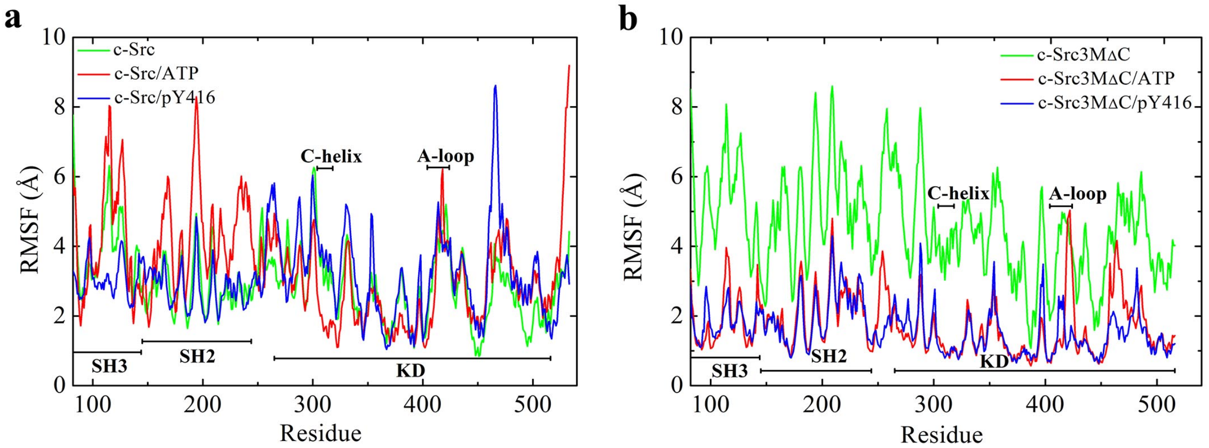

Supplementary Figure S4. Root-mean square fluctuations (RMSF, in Å) calculated for Cα atoms, averaged over the last 0.5 μs of the MD simulations for each model.

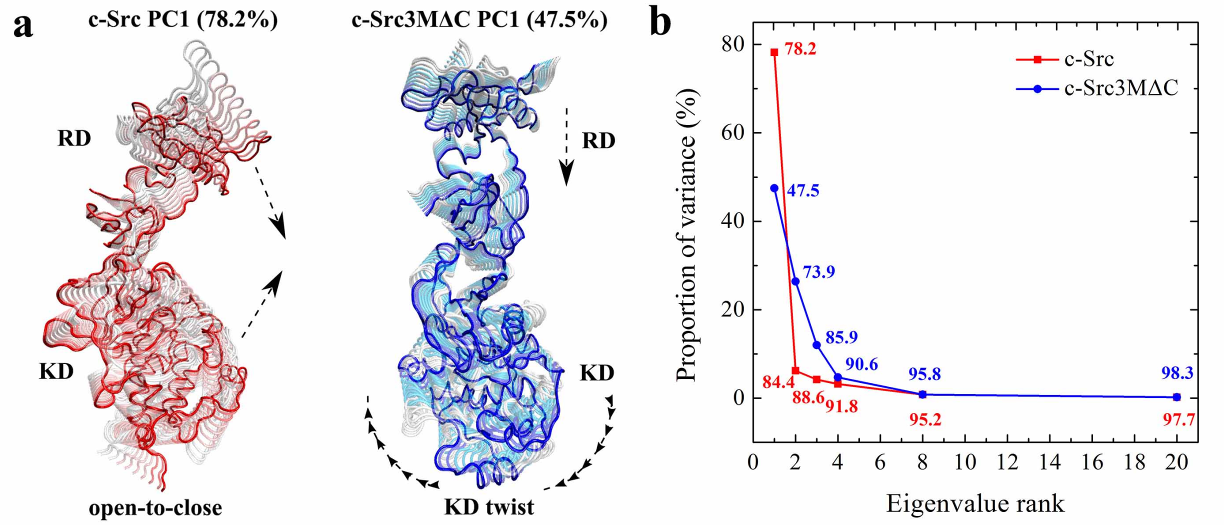

Supplementary Figure S5. (a) Principal component 1 (PC1) of c-Src and c-Src3MΔC. The arrows show the direction of motions of the regulatory domains (RD, including SH3 and SH2) and the kinase domain (KD). The motions are displayed from the first transparent structure (in white) to the last colored structure. In c-Src, the RD and the KD are involved in an open-to-close motion, while in c-Src3MΔC, a twist motion of the KD is observed. (b) Scree plots for PCA of c-Src and c-Src3MΔC.

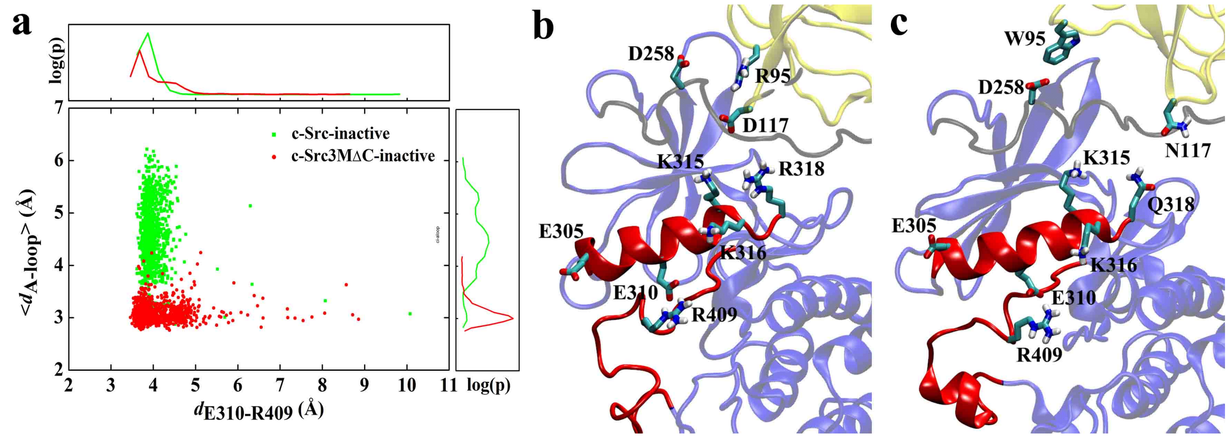

Supplementary Figure S6. (a) Conformational sampling of the central ion pair E310-R409 ($d_{\mathrm{E310\text{-}R409}}$) and the extent of the A-loop (<$d_{\mathrm{A\text{-}loop}}$>) in the inactive state of the wild type c-Src (green dots) and c-Src3MΔC (red dots). Central ion pairs in the inactive state of (b) c-Src and (c) c-Src3MΔC. The C-helix and A-loop are colored in red.

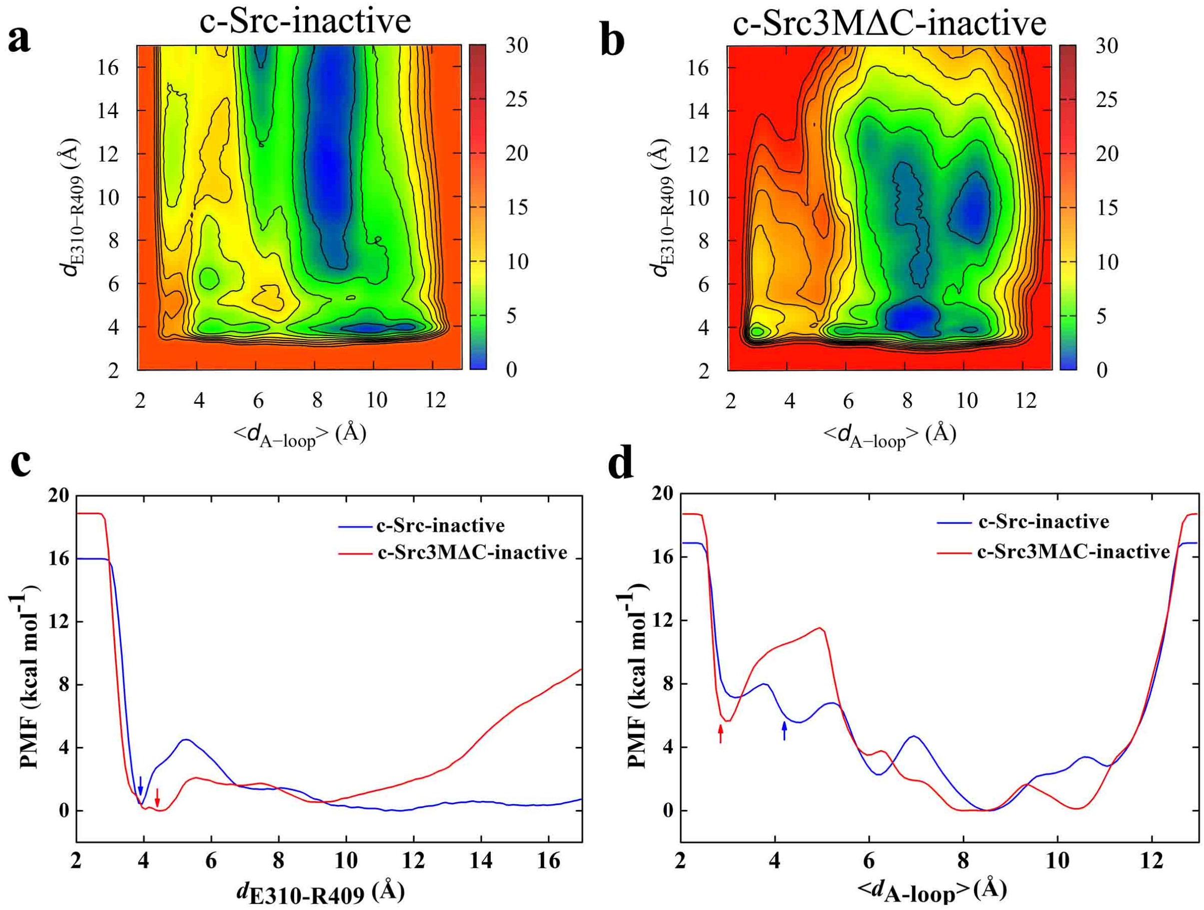

Supplementary Figure S7. Free energy landscape, ΔG (in kcal mol^{-1}) for the inactive states of (a) c-Src and (b) c-Src3MΔC obtained from 500 ns metadynamics simulations with reaction coordinates E310-R409 ($d_{E310-R409}$) and the extent of the A-loop (<d_{A-loop}>). PMF projected on $d_{E310-R409}$ and <d_{A-loop}> shown in (c) and (d), respectively. The arrows mark the starting positions of $d_{E310-R409}$ and <d_{A-loop}>.

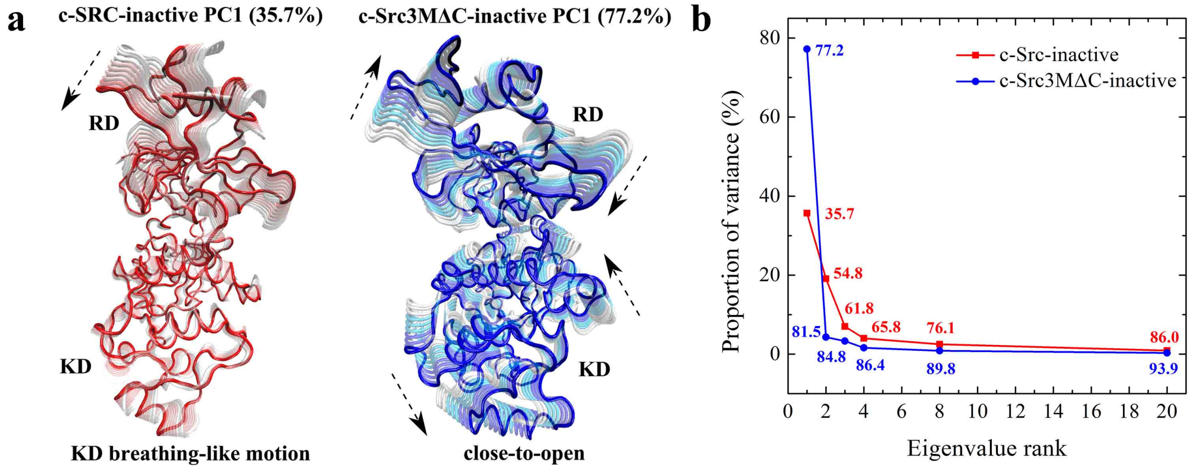

Supplementary Figure S8. A) Principal component 1 (PC1) of the inactive states of c-Src and c-Src3MΔC. The arrows show the direction of motions of the regulatory domain (RD) and the KD. The motions are displayed from the first transparent structure (in white) to the last colored structure. In the inactive sate of c-Src, PC1 comprises a breathing-like motion within the KD, while in c-Src3MΔC, the RD and the KD involve a close-to-open motion. B) Scree plots for PCA of the inactive states of c-Src and c-Src3MΔC.

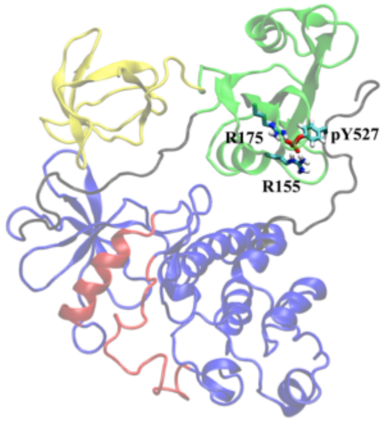

Supplementary Figure S9. Interactions between R155, R157 and pY527 connect the SH2 domain and the KD, and contribute to the rigid structure observed for the inactive state of c-Src.

Supplementary Table S1. Average interaction energies and standard deviations (±) between the protein and ATP:Mg^{2+} complex obtained from 1 μs MD simulations.

**Models**	**Energy type**	**Energy value (kcal mol^{-1})**
**c-Src - ATP:Mg^{2+}**	Total energy	-210±18
	Electrostatic interaction	-186±17
	Van der Waals interaction	-24±5
**c-Src3MΔC - ATP:Mg^{2+}**	Total energy	-442±20
	Electrostatic interaction	-429±22
	Van der Waals interaction	-13±7

Supplementary Table S2. pK_a values for residues in the active site, calculated from structures obtained after 1 μs MD simulations for c-Src and c-Src3MΔC. P and D refer to residues that are protonated and deprotonated in the pH range 0-15, respectively.

**Residue**	**pK_a in c-Src**	**pK_a in c-Src3MΔC**
**E97**	2.3	4.3
**D258**	D	3.7
**K295**	P	P
**E305**	4	4.2
**E310**	D	3.3
**K315**	13.5	10.4
**K316**	10.9	10.1
**H384**	2.7	D
**R385**	P	P
**D386**	D	D
**D404**	D	6.1
**R409**	P	14.2
**Y416**	9.9	P
**R419**	P	P

Supplementary Table S3. Average solvent accessible surface area (SASA, $Å^2$) and their standard deviations (±) of the protein backbone. The data was calculated based on the last 0.5 μs trajectory for each model for regions central to the Hsp90-dependence.

	**P-loop**	**C-helix**	**αC-β4-loop**	**E-helix**	**β1-β5-strands**
**c-Src**	478±11	934±20	814±10	1196±15	2703±43
**c-Src/ATP**	480±8	930±23	832±12	1212±17	2760±46
**c-Src/pY416**	478±7	940±15	813±11	1180±23	2784±40
**c-Src3MΔC**	477±11	929±15	814±12	1218±16	2748±50
**c-Src3MΔC/ATP**	479±9	910±16	799±21	1200±21	2769±36
**c-Src3MΔC/pY416**	473±9	951±17	830±12	1219±21	2812±42

Supplementary Table S4. Increased kinase activity and Hsp90-dependence of Src correlates with opening of the K295-E310 ion pair. The distance between K295-E310 is calculated every 1 ns. The normalized kinase activity values are obtained from Boczek *et al.*[3].

K295-E310	c-Src	c-Src3MΔC	v-Src
**Open (> 3.5 Å)**	135	728	379
**Closed (≤ 3.5 Å)**	866	273	122
**Open/ Closed**	0.156	2.667	3.107
**In vitro activity**	0.1	0.21	0.26
**In vitro activity /Hsp90-Cdc37**	0.13	0.25	0.67

Supplementary Table S5. Distances between ion pairs in the active site based on structures obtained after 1 μs MD simulations. The crystal structure (PDB ID: 1Y57) of the active state was also analyzed. The electrostatic network within this active site is perturbed due to the 3MΔC mutation, but ATP binding and pY416 recover to some extent the central interactions that stabilize the E310-K295 ion pair and the C-helix.

**Models**	**Ion pairs d < 4Å**	**Ion pairs 4 ≤ d < 5Å**	**Ion pairs 5 ≤ d < 6Å**
**1Y57**	D258-K315<br>E310-K295<br>R385-Y416	K295-D404<br>R409-Y416	R95-E305<br>E97-K315
**c-Src**	R95-D258<br>E97-K315<br>E310-K295<br>K295-D404		D386-R419
**c-Src/ATP**	E310-K295<br>K295-D404	R95-D258<br>E97-R409<br>R385-D386<br>R385-D404	R409-Y416
**c-Src/pY416**	D258-K316<br>E310-K295<br>K295-D404<br>E305-R419	R385-pY416<br>R409-pY416<br>pY416-R419	
**c-Src3MΔC**			K295-D404
**c-Src3MΔC/ATP**	E310-K295<br>K295-D404		R409-Y416
**c-Src3MΔC/pY416**	D258-K315<br>E310-K295<br>K295-D404<br>R409-pY416	E305-R409<br>R385-pY416<br>pY416-R419	

Supplementary Table S6. The simulation systems of c-Src and c-Src3MΔC explored by classical molecular dynamics (MD) simulations and metadynamics (MT) simulations.

Starting structure	Src	Cofactor	pY416	MD	MT
**1Y57 Active state**	c-Src	--	-	1 μs	0.5 μs
		ATP	-	1 μs	--
		--	+	1 μs	0.5 μs
	c-Src3MΔC	--	-	1 μs	0.5 μs
		ATP	-	1 μs	--
		--	+	1 μs	0.5 μs
	c-Src3MΔC Protonated D404	--	-	0.5 μs	--
	v-Src	--	-	0.5 μs	--
**2SRC Inactive state**	c-Src	--	-	1 μs	0.5 μs
	c-Src3MΔC	--	-	1 μs	0.5 μs

Total simulation time: 12 μs

SI References:

1. Xu, W., Doshi, A., Lei, M., Eck, M.J. & Harrison, S.C. Crystal structures of c-Src reveal features of its autoinhibitory mechanism. *Mol. Cell* **3,** 629-638 (1999).
2. Young, M.A., Gonfloni, S., Superti-Furga, G., Roux, B. & Kuriyan, J. Dynamic coupling between the SH2 and SH3 domains of c-Src and Hck underlies their inactivation by C-terminal tyrosine phosphorylation. *Cell* **105,** 115-126 (2001).
3. Boczek, E.E. et al. Conformational processing of oncogenic v-Src kinase by the molecular chaperone Hsp90. *Proc. Natl. Acad. Sci. U.S.A.* **112,** E3189-E3198 (2015).
4. Meng, Y. & Roux, B. Locking the active conformation of c-Src kinase through the phosphorylation of the activation loop. *J. Mol. Biol.* **426,** 423-435 (2014).
